# The Future of PSMA-Targeted Radionuclide Therapy: An Overview of Recent Preclinical Research

**DOI:** 10.3390/pharmaceutics11110560

**Published:** 2019-10-29

**Authors:** Eline A.M. Ruigrok, Wytske M. van Weerden, Julie Nonnekens, Marion de Jong

**Affiliations:** 1 Dept. of Radiology and Nuclear Medicine, Erasmus MC, 3015 GD Rotterdam, The Netherlands; 2 Dept. of Experimental Urology, Erasmus MC, 3015 GD Rotterdam, The Netherlands; 3 Dept. of Molecular Genetics, Erasmus MC, 3015 GD Rotterdam, The Netherlands; 4 Oncode Institute, Erasmus MC, 3015 GD Rotterdam, The Netherlands

**Keywords:** prostate specific membrane antigen, prostate cancer, targeted radionuclide therapy

## Abstract

Prostate specific membrane antigen (PSMA) has become a major focus point in the research and development of prostate cancer (PCa) imaging and therapeutic strategies using radiolabeled tracers. PSMA has shown to be an excellent target for PCa theranostics because of its high expression on the membrane of PCa cells and the increase in expression during disease progression. Therefore, numerous PSMA-targeting tracers have been developed and (pre)clinically studied with promising results. However, many of these PSMA-targeting tracers show uptake in healthy organs such as the salivary glands, causing radiotoxicity. Furthermore, not all patients respond to PSMA-targeted radionuclide therapy (TRT). This created the necessity of additional preclinical research studies in which existing tracers are reevaluated and new tracers are developed in order to improve PSMA-TRT by protecting the (PSMA-expressing) healthy organs and improving tumor uptake. In this review we will give an overview of the recent preclinical research projects regarding PCa-TRT using PSMA-specific radiotracers, which will give an indication of where the PSMA-TRT research movement is going and what we can expect in future clinical trials.

## 1. Introduction

Prostate cancer (PCa) is a major public health problem; approximately 450,000 men have been diagnosed with PCa in Europe in 2018 [1]. Although the 5 year survival rate for local and regional PCa is 99%–100%, this rate drops to 30% for patients with metastasized PCa [2]. Currently, prostate-specific antigen (PSA) level measurements, digital rectal examination, and Gleason score grading are being used for the diagnosis and staging of PCa. Current treatment options for PCa patients involve surgery and radionuclide therapy for local disease, while hormone therapy, anti-androgen therapy, and chemotherapy are therapeutic options for advanced disease [3]. Patients at high risk for metastatic disease, based on high plasma PSA, but with negative prostate biopsy or patients with rising PSA levels after local treatment with curative intent, undergo additional staging using computed tomography (CT), whole body magnetic resonance imaging (MRI), or ^18^F choline-based positron emission tomography (PET)/CT. These imaging techniques unfortunately have limited sensitivity for detecting small metastatic lesions [4].

In 1987, the type II transmembrane enzymatic protein prostate specific membrane antigen (PSMA) was discovered as a novel antigenic marker in PCa cells and serum of PCa patients [5]. PSMA is overexpressed in 90%–100% of PCa cases and the level of PSMA expression is highly correlated with disease progression, with high PSMA expression being found in hormone-resistant tumors and metastases [6,7,8]. The overexpression of PSMA on (metastasized) PCa cells makes it an excellent target for imaging and therapy using PSMA-targeting tracers. These tracers can be labeled with different varieties of radionuclides for diagnostic (e.g., positron emitter gallium-68) or therapeutic purposes (e.g., beta-particle emitter lutetium-177 or alpha-particle emitter actinium-225). The radiolabeled tracers are administered intravenously and will accumulate at the tumor sites due to their PSMA-targeting properties. Radioactive decay of the alpha and beta particle emitting radionuclides can induce DNA damage which ultimately may lead to cancer cell death. The excellent potential application of PSMA-targeting tracers resulted in the development of a multitude of different tracers for diagnostic and therapeutic purposes.

The high interest in PSMA-targeted radionuclide therapy (TRT) led to a quick movement from preclinical research to clinical trials, which are showing very promising results. However, next to PCa cells, expression of PSMA is also found in healthy prostate tissue, the small intestine, central nervous system, proximal renal tubules, and the salivary and lacrimal glands [9,10,11]. These PSMA-expressing organs show uptake of almost all PSMA-targeting radiotracers, which might cause toxicity. Especially when using an alpha particle-emitting radionuclide (e.g., actinium-225), severe xerostomia can occur, which can have an enormous negative impact on the quality of life of the patients [12].

The high number of developed PSMA-specific tracers and their almost immediate transit to clinical trials offered a major advantage in the treatment of metastasized PCa patients. However, as described above, many tracers showed uptake in healthy organs causing radiotoxicity and not all patients responded to PSMA-TRT [13]. This created the necessity of additional preclinical research studies in which existing tracers were reevaluated and improved tracers were developed.

**Figure 1:**
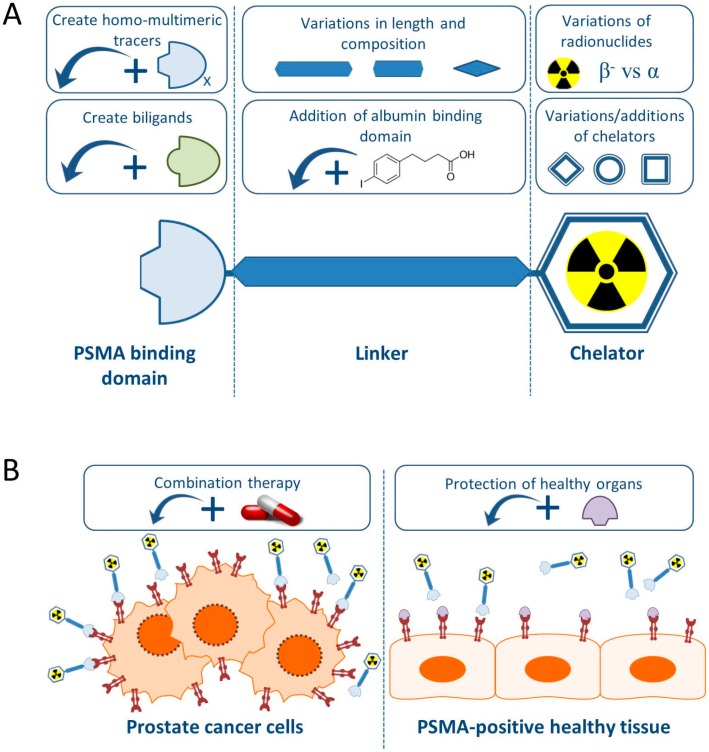
Schematic overview of preclinical research regarding the improvement of prostate specific membrane antigen-targeted radionuclide therapy (PSMA-TRT). (**A**) The basic building blocks of a PSMA-targeting small molecule inhibitor consist of a PSMA-binding domain, a linker and a chelator which can be labeled with various radionuclides. For each part of the tracer, additions or variations are being preclinically studied and described in this review. (**B**) Next to alterations to the targeting molecule, increasing the therapeutic impact on the tumor (left) is tried by combining PSMA-TRT with for instance androgen deprivation therapy. Protection of the healthy PSMA-expressing tissue (right) is studied by blocking the PSMA-molecule with for instance 2-PMPA.

In this review we will give an overview of the recent preclinical research projects regarding PCa TRT using PSMA-specific radiotracers, with special emphasis on small molecule PSMA inhibitors. Figure 1 summarizes the preclinical approaches discussed in this review.

### PSMA-Targeting Small Molecule Inhibitors

Generally, PSMA-targeting small molecule inhibitors consist out of a PSMA binding domain, a linker and a domain to which radionuclides can be labeled, such as the chelator 1,4,7,10-tetra-azacycloododecane-*N*,*N*′,*N*″,*N*‴-tetraacetic acid (DOTA) or 1,4,7,10-tetraazacyclododececane,1-(glutaric acid)-4,7,10-triacetic acid (DOTAGA) (Figure 1).

The development of PSMA-targeting small molecule inhibitors skyrocketed from the moment it was discovered that PSMA is chemically identical to NAALADase (*N*-acetyl-alpha-linked-acidic-dipeptidase), a neurotransmitter catalyzer, and to folate hydrolase 1 (FOLH-1), which is expressed in the jejunal brush border [14]. Already existing inhibitors of NAALDase could therefore be used as PSMA-targeting tracers. Furthermore, numerous other small molecule inhibitors have been derived and some of those are currently being studied in clinical trials (Table 1). An elaborate overview of PSMA-targeting tracer development in the past two decades can be found in earlier published reviews [15,16,17,18,19,20,21].

PSMA-targeting small molecule inhibitors can be divided into three types; urea-based, phosphorus-based, and thiol-based. The urea-based compounds have shown superior binding affinity for PSMA compared to the other two types. Hence, most (novel) PSMA-specific small molecule inhibitors carry the urea-based binding domain [22]. These urea-based PSMA binding motifs are present in three forms: glutamate-urea-lysine (glu-urea-lys), glutamate-urea-glutamate (glu-urea-glu), or glutamate-urea-cysteine (glu-urea-cys) [23].

Today, PSMA-617 and PSMA-I&T are the tracers most often applied to PSMA-TRT in preclinical research and clinical trials. Both share the same glu-urea-lys–PSMA binding motif and their DOTA/DOTAGA chelators enable labeling with therapeutic radionuclides [24,25].

## 2. Improving PSMA-Targeting Small Molecule Inhibitors

Despite the promising results of numerous clinical trials with PSMA-targeting small molecule inhibitors labeled with beta particle-emitting or alpha particle-emitting radionuclides, a necessity for optimization of these tracers has become prominent because of the healthy organ toxicity discussed above [40,41]. Therefore, the majority of preclinical research is focused on developing and/or adjusting PSMA-targeting tracers to make them safer and better. Different strategies to optimize the tissue distribution characteristics of the current PSMA-targeting tracers are being applied, for instance by adding an albumin binding domain or by linker modifications, intending to prevent the uptake in the salivary glands and kidneys and increase tumor binding.

### 2.1. Addition of an Albumin Binding Domain

A possible method to reduce toxicity on healthy organs during PSMA-TRT would be to decrease the injected dose of radiolabeled tracer. However, this might probably lead to a reduction of anti-tumor efficacy. This problem could be overcome by elongation of the circulation time of the radiolabeled tracers, most likely leading to an increase in tumor uptake. In this way, a lower quantity of the radiolabeled tracer can be injected while reaching the same level of tumor targeting [42].

The circulation time of rapidly cleared molecules can effectively be increased by adding a plasma protein binding domain [43]. Albumin is an ideal plasma protein target as it is the most abundant protein in the blood and has a half-life of 19 days in the human blood circulation [44,45]. Next to the advantage of the increased circulation time, addition of an albumin binding domain is suggested to have extra advantages. For instance, due to the increased size of the conjugated tracer, the chance that the radiolabeled compound can accumulate in the tumor increases due to the leaky tumor vasculature versus the impermeable vascular walls of the healthy tissue [46]. Furthermore, it is suggested that tumor uptake of albumin conjugated tracers will increase because of the overexpression in the tumor environment of albumin-binding proteins such as secreted protein acidic and rich in cysteine SPARC and the glycoprotein 60 (gp60) receptor, which play crucial roles in angiogenesis and capillary permeability, respectively [47].

The principle of adding an albumin binding domain to increase the circulation time and decrease normal tissue binding of radiolabeled compounds was first proven by Müller et al., who measured the binding of [^177^Lu]Lu-cm09, a folate ligand with an albumin binding domain [48]. This research showed a 3× higher in vivo tumor uptake 24 h post injection (p.i.) and a 2.5× lower kidney uptake 4 h p.i. of the folate ligand with albumin binding domain compared to [^177^Lu]Lu -EC0800, the same folate ligand without albumin binding domain. Subsequently, several research groups conjugated the 4-(*p*-iodophenyl)butyric acid as an albumin binding domain to PSMA-targeting small molecule inhibitors tracers [37,38,49,50,51]. These PSMA-albumin binding tracers (CTT1403, RPS-027, RPS-063, DOTA-PSMA-ALB-02 and HTK01169) all successfully showed increased circulation-half-lives and tumor uptake. However, unlike the findings of Müller et al. with the folate ligands [48], the addition of this albumin binding domain also dramatically increased the renal uptake for these tracers during in vivo murine experiments, which may be caused by the increased circulation time. Similar results were presented by Wang et al. who coupled the truncated Evans blue (tEB) albumin binding domain to their PSMA-targeting compound (DOTA-EB-MCG) [46].

Follow-up studies aimed to exploit the increased tumor uptake and tried simultaneously to prevent the increased renal uptake. For this, Umbricht et al. compared the 4-(*p*-iodophenyl)butyric acid with a *p*-(tolyl)-moiety as an albumin binding domain conjugated to the DOTA-PSMA-617 compound, creating PSMA-ALB-53 and PSMA-ALB-56, respectively [52]. The *p*-(tolyl)-moiety is a weaker albumin binder compared to PSMA-ALB-53 and showed a faster in vivo renal clearance when labeled with lutetium-177. Interestingly, [^177^Lu]Lu-PSMA-ALB-56 demonstrated a higher tumor uptake compared to [^177^Lu]Lu-PSMA-ALB-53 which led to a 3× higher tumor-to-kidney ratio of [^177^Lu]Lu-PSMA-ALB-56 compared to [^177^Lu]Lu-PSMA-ALB-53. In vivo therapy studies showed an increased survival benefit of [^177^Lu]Lu-PSMA-ALB-56 compared to [^177^Lu]Lu-PSMA-617, probably due to its higher tumor uptake. However the tumor-to-kidney ratio of [^177^Lu]Lu-PSMA-617 was 3× higher compared to [^177^Lu]Lu-PSMA-ALB-56. The use of a weaker albumin binder might be an ideal compromise leading to increased tumor uptake due to the albumin binding, although with enhanced risk for renal toxicity.

Summarized, increased circulation half-life of a PSMA-targeting radiotracer by addition of an albumin binding domain successfully increased tumor uptake. However, the concomitant significant increase in renal uptake is unfavorable and should be prevented. Moreover, the longer circulation time of these adapted tracers might also lead to increased bone marrow toxicity as well as toxicity in the salivary and lacrimal glands of PCa patients, which has a major negative impact on the quality of life of these patients. Table 2 gives an overview of the developed albumin binding compounds described here.

### 2.2. Linker and Chelator Modifications

Other structural modifications of small molecule inhibitors have been reported in attempts to improve the therapeutic efficacy and tumor-to-background ratios. One option is to change the linker molecule in between the PSMA binding domain and chelator, since there is increasing evidence that PSMA-binding is not only caused by the PSMA-binding motif in these molecules, but also the hydrophobicity, charge, and overall structure is thought to influence PSMA binding efficacy [55]. Furthermore, it is suggested that binding of PSMA-targeting tracers to, for instance, the salivary glands might also be due to the hydrophobic character of the linker [56]. Therefore, modification of the linker can have significant impact on binding affinity, biodistribution, and overall pharmacokinetic properties of PSMA-targeting small molecule inhibitors [57].

Benesova et al. first showed that modifying the linker between the PSMA-binding domain and the DOTA chelator can significantly influence the pharmacokinetic properties by optimizing the linker moiety when creating PSMA-617 small molecule inhibitors [26]. Eighteen chemically different PSMA-targeting small molecule inhibitors were developed by altering the configuration of the linker. This resulted in significant changes in the pharmacokinetic properties of the tracers, thereby providing evidence that linker modification can have an impact on pharmacokinetic properties. The changes in these linkers did however not lead to superior tumor-targeting and pharmacokinetic properties compared to PSMA-617. By experimenting with these different linkers, the researchers generated knowledge of the binding effects of structural changes in PSMA-targeting tracers and proposed that this may lead to a more rational and structure-aided design and synthesis of novel PSMA-targeting small molecule inhibitors [55].

Schmidt et al. conjugated a carbohydrate to the linker of PSMA-I&T as a strategy to reduce the unwanted renal accumulation. They successfully showed the feasibility of the conjugation of such a bulky carbohydrate moiety to PSMA-I&T. This conjugation had no negative effect on the target affinity; however, the in vitro internalization rate decreased considerably. Furthermore, no beneficial effects were shown of the conjugated carbohydrate moiety in vivo [58]. Another linker modification to a PSMA small molecule inhibitor suitable for therapy was tested by Kuo et al. The authors replaced the 2-naphthylalanine (2-Nal) amino acid of the linker of PMSA-617 with either 2-indanylglycine (Igl) or 3,3-diphenylalanine (Dip), which are close analogs of 2-Nal. Doing so, the researchers hypothesized that these changes would lead to a stronger interaction with the PSMA target. However, the substitution with Igl or Dip led to a ~5- and 21-fold decrease, respectively, of in vitro binding affinity. The compound with the Igl substitution (HTK01166) showed similar in vivo tumor binding as PSMA-617, however an almost 5× higher renal uptake. The Dip replacement (HTK01167) on the other hand, led to a 6× lower kidney binding and a 2× lower tumor binding. This indicates the potential of HTK01167 as a theranostic tracer with lower tumor-background ratios and warrants further development [59].

Kelly et al. tried to optimize the binding characteristics of their developed albumin binding PSMA-tracer RPS-63 via an increased polyethylene glycol (PEG) spacer length between the PSMA binding domain and the albumin binding domain. The newly synthesized RPS-072 showed a higher tumor uptake compared to RPS-63 and PSMA-617. The researchers argued that the increased linker size reduced the retention of the radioligand by increasing its clearance before the tubular reabsorption. However, besides increased tumor uptake, renal uptake of RPS-072 increased as compared to PSMA-617 [49].

These examples of linker modifications support the hypothesis that linker structure has an influence on tracer pharmacokinetic properties and show the feasibility of alternating the structure. However, none of the previous discussed modifications has led to an increased tumor binding and a decrease in the binding to the healthy PSMA-expressing organs in comparison to PSMA-617 or PSMA-I&T, respectively.

In addition to altering linker structure, the chelating moiety is also interchangeable and can have a significant effect on the pharmacokinetics [60,61]. For example, replacing the DOTA chelator of PSMA-617 with a CHX-A’’-DTPA conjugate, Wüstemann et al. showed higher in vitro internalization rate facilitated by the CHX-A’’-DTPA chelator compared to PSMA-617. Furthermore, a progressive long-term accumulation in LNCaP tumors in vivo was observed, as the CHX-A’’-DTPA conjugate uptake increased until 3 h p.i. The researchers suggested that this was due to the higher internalization rate that they measured in vitro. However, it must be noted that the DOTA version of PSMA-617 has an approximately 2× higher tumor uptake in vivo [57].

Next to changing the chelator moiety of PSMA-targeting tracers, also the addition of an extra chelator is being investigated as a method to label compounds with both therapeutic and diagnostic radionuclides. For instance, Wurzer et al. synthesized such a dual-nuclide labeled tracer (gallium-68 for PET and bismuth-213 for therapy) and proved the concept of in vivo monitoring the biodistribution via PET and therefore enabling precise therapeutic dosimetry. Limitation of this approach is that radionuclides with matching half-lifes must be used in order to correctly determine dosimetry [62]. In line of this research, Wester et al. proposed the idea for a PSMA radiohybrid (rhPSMA) tracer that consists of a PSMA binding domain, a radiometal binding chelator and an extra functional group for covalent fluorine-18 labeling. This rhPSMA tracer will be suitable for PET imaging when labeled with fluorine-18 and the chelator is filled with a cold/non-radioactive metal, like lutetium-175. When using this tracer for therapy, the rhPSMA tracer can be labeled with lutetium-177 and fluorine-19. Using this approach, the tracer used for diagnostic PET imaging and the tracer used for therapy are chemically 100% identical. These rhPSMA tracers therefore will have the same binding affinities, selectivities, lipophilicities, and pharmacokinetics. This has the advantage of pretherapeutic PET-imaging providing valid quantitative biodistribution data and dosimetry, which can be used for personal therapeutic intervention [25]. 

Thus, the structure of the linker and the type of chelator can have an effect on the overall binding properties of PSMA-specific agents. Experiments with structural differences in these tracers gives more insight in PSMA-binding and internalization both in PCa and healthy PMSA-expressing organs and further research is warranted.

## 3. Development of Multi- and Bi-Ligands

In order to increase the binding affinity of PSMA-specific tracers, some groups have focused on the development of homo-multimeric tracers, which contain more than one PSMA-binding domain [62,63,64]. These multivalent PSMA-specific tracers have shown promising results in vitro, increased binding affinity, and in vivo, increased tumor retention. However, these tracers have not been introduced in the clinic for therapeutic purposes yet.

Looking beyond PSMA, PCa cells also express other target proteins that are suitable for TRT. Combining a PSMA-targeting tracer with a tracer targeting another PCa specific target might increase uptake in PCa lesions and show enhanced theranostic properties. The probability of a double targeting tracer, or biligand, to bind to PCa is larger especially of value for tumors with a high heterogeneity in expression of different targets [65,66]. However, combination of two targeting molecules might also have the drawback of interfering with binding to the PSMA target or increasing binding on PSMA-expressing or other healthy organs. Furthermore, increasing the size of PSMA-targeting tracers may have negative effects on the pharmacokinetic properties. 

### 3.1. Targeting PSMA and Hepsin/Integrins

Various bi-ligands have been developed in which PSMA-targeting tracers are combined with another specific binding domain. For example, Subedi et al. combined a Lys-urea-Glu moiety PSMA-binding scaffold with a binding domain targeting hepsin, a membrane serine protease that is highly upregulated in PCa [67]. Conjugated with the optical dye SulfoCy7, this compound showed higher binding in PSMA- and hepsin-expressing tumors compared to tracers that target PSMA alone, which suggests that this PSMA/hepsin heterodimer might be useful in increasing sensitivity of PCa diagnostic imaging [68]. A PSMA-hepsin heterodimer suitable for radionuclide therapy and the corresponding healthy organ uptake has not been investigated yet.

Another example that proves the feasibility of creating PSMA heterodimers is provided by Shallal et al. Here, the authors combined the PSMA binding domain with a domain targeting α_v_β_3_ integrin reported to be highly upregulated in PCa. The researchers were able to prove in vitro that the PSMA/α_v_β_3_ integrin heterodimer could bind to both targets; however, the binding affinity was much lower compared to DCIBzL, a high affinity PSMA binding agent. This compound is being taken further into preclinical testing by performing quantitative biodistribution assays and imaging in vivo [69].

### 3.2. Targeting PSMA and the Gastrin-Releasing Peptide Receptor

Another relevant PCa target is the gastrin-releasing peptide receptor (GRPR). GRPR is a transmembrane G-protein coupled receptor which is overexpressed in 84% of PCa cases, as well as in other types of cancer, such as breast cancer and small cell lung cancer [70,71]. Beside expression in tumor tissue, GRPR is expressed in various healthy tissues, such as breast, pancreas, prostate, and lung [72]. While PSMA expression increases as the disease progresses, GRPR is already overexpressed in high levels in early PCa stages [71]. Therefore, combined targeting of both PSMA and GRPR could lead to a high tumor uptake during all disease stages. 

In 2014, the first PSMA/GRPR heterodimer ((DUPA-6-Ahx-(^64^Cu-NODAGA)-5-Ava-BBN(7–14)NH_2_)) was created by Bandari et al. with the aim to improve PCa imaging [73]. Although this compound did not show superior imaging properties compared to existing mono-targeting PSMA-tracers, the study did show that constructing such a PSMA/GRPR targeting tracer was feasible. Rapidly, several PSMA/GRPR targeting heterodimers were developed and preclinically tested [66,70]. These PSMA/GRPR bi-ligands all showed specific PSMA and GRPR PCa tumor targeting in vitro and in vivo. However, the combination of the two targeting domains also combined with undesired high uptake in the kidneys, spleen, and pancreas. Alterations in the linker compositions by increasing the number of charged amino acids resulted in a significant decreased uptake in the kidneys and spleen. Unfortunately, it also resulted in increased uptake of the GRPR-positive pancreas [65].

Because research models of PCa used in the previously described research projects only overexpress either PSMA or GRPR, but not both targets simultaneously, it is yet impossible to show that combining these targets will have a synergistic or additive tumor targeting effect. Abouzayed et al. did use an in vivo tumor model that both expresses PSMA and GRPR, PC3-PIP, while studying their GRPR/PSMA-heterodimer [^125^I]I-BO530 [74]. [^125^I]I-BO530 showed long activity retention in these PC3-PIP tumors however, high uptake in the kidneys led to unfavorable low tumor-to-kidney ratios of 1.2 ± 0.3 at 24 h p.i. which, so the researchers argue, could lead to an unacceptably high renal radiation dose. Furthermore, the researchers did not compare [^125^I]I-BO530 to a single PSMA-targeting tracer (for instance PSMA-617), which is necessary in order to prove synergistic advantages of the PSMA-GRPR heterodimer. 

These studies showed that it is feasible to create heterodimer tracers that target both PSMA and GRPR. However, it must be noted that with the addition of the GRPR moiety, the salivary glands and kidney toxicity caused by PSMA-TRT might be accompanied by the GRPR-TRT toxicity in GRPR-expressing pancreas. This must be carefully considered before introducing these bi-ligands to the clinic.

## 4. Varying Radionuclides for PSMA-TRT

**Figure 2:**
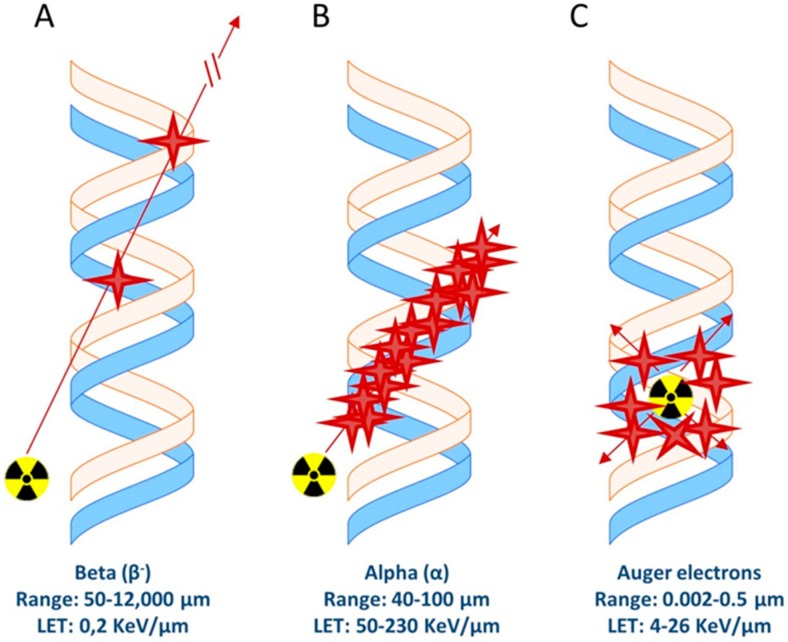
Schematic representation of ionization events caused by (**A**) beta particles, (**B**) alpha particles, and (**C**) Auger electrons.

There are different types of radionuclide emissions that can cause cellular/DNA damage and therefore can be used for therapeutic purposes; Auger/conversion electrons and beta- (β^−^) and alpha (α) emission (Figure 2). These emissions have different linear energy transfer (LET, in Kev/um) values ranging from a few eV for Auger/conversion electrons up to MeV for alpha particles (Table 3). Importantly, the range of these particles varies. While alpha and beta radiation reaches a range of 40–100 µm and 50–12,000 µm, respectively, Auger/conversion electrons have a very short range of only 0.002–0.5 µm [75]. Delivery of the radiolabeled compounds which carry a radionuclide with such a short range must therefore be very close to or directly in/near the cell nucleus in order to cause DNA damage.

### 4.1. Beta-Emitters

Today, lutetium-177 is most frequently used for PSMA-TRT in the (pre)clinic. With a mean range of 670 µm and energies of 0.1–2.2 MeV of the beta particles, it is an ideal radionuclide for treatment of micro-metastases. Lutetium-177 also emits γ-rays during its decay, enabling single photon emission computed tomography (SPECT) imaging for treatment efficacy predictions. Despite these favorable properties, clinical studies have shown that approximately 30% of PCa patients did not respond to lutetium-177 PSMA-TRT [76,77]. Therefore, many (pre)clinical research projects focus on experimenting with different radionuclides to reach a higher therapeutic efficiency.

Alternative potent beta-emitting radionuclides are copper-67, scandium-47 and terbium-161. These radionuclides have an energy emission comparable to that of lutetium-177 [78]. Müller et al. studied the potency of terbium-161 for PSMA-TRT, which, besides beta decay, also emits a considerable amount of Auger/conversion electrons, leading to a possible increase in total absorbed dose compared to lutetium-177 [79]. In vivo biodistribution assays revealed comparable biodistribution profiles for PSMA-617 labeled with lutetium-177 or terbium-161. In vitro, [^161^Tb]Tb-PSMA-617 had a significantly higher therapeutic efficiency on PC3-PIP cells compared to [^177^Lu]Lu-PSMA-617. An in vivo therapy study comparing [^177^Lu]Lu-PSMA-617 and [^161^Tb]Tb-PSMA-617 is currently lacking, indicating that additional pre-clinical research is needed to test the potential of these tracers.

### 4.2. Alpha-Emmiters

Targeted alpha therapy (TAT) has become of high interest for PSMA-TRT. Alpha particles have much higher LET and a shorter range compared to beta-particles, which can lead to several ionization events in close proximity to each other within the DNA causing so-called alpha-tracks [80,81]. Therefore, in comparison to beta decay, alpha decay can cause a high quantity of DNA double strand breaks in a short range and holds the promise of a higher level of induced DNA damage per cell. Actinium-225, lead-212, thorium-227, bismuth-213, and astatine-211 are alpha emitting radionuclides that are being explored (pre)clinically for PSMA-TAT [54,80,82,83]. These studies all prove the increased therapeutic effectiveness of alpha-radiation compared to beta-radiation. Mice treated with a single dose of [^225^Ac]Ac-RPS-074 (148 kBq) even showed total remission underlining the potential of actinium-225 for PSMA-directed radionuclide therapy [54]. The increase in therapeutic efficiency by the use of alpha-radiation, however, may also lead to an increase in (late) onset of toxicity. Preclinical in vivo studies did not address these long-term toxicity effects, however, several clinical PSMA-TRT studies with actinium-225 reported irreversible damage to lacrimal and salivary glands, causing xerostomia [84]. Xerostomia is one of the major concerns for PSMA targeting therapy as it has a major negative impact on the quality of life of the patients and should therefore be prevented.

Lead-212 emits beta-particles and decays into bismuth-212, which emits alpha-particles. Lead-212 is suggested as a potent alternative for actinium-225 because of its shorter half-life (10 h vs. 10 days) and fewer alpha emitting daughters (1 vs. 3), thus potentially lower normal organ toxicity risks [85,86]. Banerjee et al. recently published their proof-of-concept of a novel [^212^Pb]Pb-labeled L2, a PSMA low molecular weight compound [86]. In vivo murine dose-dependent tumor growth inhibition was demonstrated, resulting in increased survival benefit when compared to [^177^Lu]Lu-PSMA-617. Unfortunately, long term renal toxicity was observed in healthy non-tumor bearing mice after a single administration of [^212^Pb]Pb-L2, revealing the kidneys as dose-limiting organs in mice.

Taken together, various radionuclides with different characteristics have been tested in preclinical experiments for their efficacy and toxicity for PSMA-TRT. Currently, lutetium-177 is most frequently used in the (pre)clinic, followed by actinium-225. Further preclinical and clinical research is required to define the most optimal tracer-radionuclide combination which combines the highest therapeutic efficiency with the lowest toxicity. Further description of current PSMA-TAT research can be found in the review of Chakravarty et al. [40].

## 5. Enhancement of Therapy Effect

So far, we have elaborately discussed the approaches used to develop novel or adjust existing tracers in order to increase the therapeutic efficacy of PSMA-TRT. The variations in tracer structures and combinations are in theory endless and thus might result in the development of a magnitude of different PSMA-targeting tracers. Another strategy in order to reach a higher therapeutic efficacy may be combining PSMA-TRT with other (existing) therapies for PCa [87,88]. For example, using other agents to increase the PSMA expression on tumor cells or to increase the radiosensitivity of the tumor could significantly increase PSMA-TRT outcome.

Chemical radiosensitizers are compounds that are intended to increase the level of damage induced by ionizing radiation [89]. This combination strategy has been widely researched and clinically adapted for PCa external beam radiation therapy (EBRT) and has shown promising increases in therapeutic efficacy [90]. Radiosensitizing agents can be divided into three working mechanism categories; agents that dysregulate the cell cycle, agents that inhibit DNA damage repair, and agents that cause oxygenated stress.

Radiosensitization in combination with PSMA-TRT has not yet thoroughly been investigated. Tesson et al. combined and compared the therapeutic effect of PSMA-TRT with several radiosensitizing compounds in a preclinical setting [91]. When combining [^131^I]I-MIP-1095 with either an oxidizing agent (disulfiram), p53 pathway inhibitor (nutlin-3), proteasome inhibitor (bortezomib), or a PARP-1 inhibitor (olaparib), the researchers found an increased growth inhibition on LNCaP spheroids in vitro.

Other possible radiosensitizers to combine with PSMA-TRT are androgen deprivation therapy (ADT) or anti-androgens, such as enzalutamide and apalutamide. These treatments are already being used for the treatment of locally advanced PCa and non-metastatic castration-resistant PCa [92,93,94]. Various clinical studies have reported an overall survival benefit of ADT when combined with EBRT [95,96,97]. Recent preclinical work has revealed that enzalutamide and apalutamide act as radiosensitizers of EBRT via downregulation of the DNA double strand break repair capacity of PCa cells [98,99,100]. PSMA expression is thought to be regulated by androgens although reports showed conflicting results. While several studies have reported an increase in the level of PSMA expression in PCa cell-lines and in tumor tissue of patients after ADT [101,102,103], others describe a downregulation of PSMA expression caused by ADT [104,105]. Preclinical in vivo studies have so far been unsuccessful in showing an additive therapeutic effect on the tumor growth of this combination therapy [106]. Clearly, the added value of ADT in combination with PSMA-TRT and its mechanism of action is unresolved [107].

Alternative combination options, for instance EBRT, immunotherapy or other radiosensitizers that are proposed for increasing PSMA-TRT efficacy need to be further explored. Clearly, more preclinical research is warranted to establish potential synergistic or additive effects of combination therapy with PSMA-TRT.

## 6. Protection of PSMA Expressing Kidneys and Salivary Glands

One of the major disadvantages of PSMA-TRT is the high accumulation of the radiolabeled tracers in healthy PSMA-expressing organs, the kidneys and salivary glands. Despite the high uptake in the kidneys, there is very little reported on renal toxicity after PSMA-TRT. This could be explained by the fact that most patients receive PSMA-TRT as a last-option therapy and therefore follow-up is not long enough to detect (late) onset of renal toxicity [108]. However, when PSMA-TRT will be used for patients that are in an early stage of (high risk) PCa with (oligo)-metastatic disease, renal toxicity might become of major concern [109].

PSMA-TAT with actinium-225 has been reported to cause severe and irreversible salivary gland toxicity leading to xerostomia [110]. Xerostomia can have a major impact on the quality of life of the PCa patients that receive the potentially life-elongating PSMA-TRT: in a recent study, 10% of the patients chose to discontinue their treatment for this reason [84]. Clinical studies have investigated various strategies in order to find a method to protect the salivary glands. Amongst others, external cooling of the salivary glands with ice-packs, saline irrigation, steroid injection, botulinum toxin injections, and oral administration of folic polyglutamate tablets have been evaluated, but with no significant decrease in salivary gland uptake of PSMA-targeting tracers or in too few patients to enable statistical analysis [25,108,111,112].

There clearly is an urgent need to conduct preclinical research to test strategies to protect the salivary glands from damage during PSMA-TRT. However, the lack of a proper in vivo research model for salivary gland PSMA-TRT damage is a major drawback and relatively few preclinical studies have been published on this topic. Mice have high levels of PSMA expression in the brain, kidneys, and salivary glands, at similar levels to that observed in humans. However, in contrast to humans, mice have very low to no PSMA expression in the prostate and jejunum. Furthermore, despite the high PSMA expression in murine salivary glands, biodistribution assays with various PSMA-targeting tracers did not show any, or very low salivary gland uptake, while renal uptake is similar or even higher in mice compared to humans [113,114,115,116]. This difference in PSMA expression might be explained by the difference in PSMA amino acid sequence between humans and mice (91% similarity). Furthermore, the PSMA structures in murine kidneys and salivary glands have been reported to differ [117,118]. Finally, the high PSMA binding in the salivary gland in humans may not only be due to PSMA expression, but might also be the result of non-specific binding [119,120].

Roy et al. aimed to identify an animal model that resembles human salivary gland uptake of PSMA-TRT and compared the salivary glands uptake of [^18^F]F-DcFPYL in NCR-nu/nu athymic female mice and F344/SAS Fischer female rats [121]. Biodistribution assays revealed a 2- to 3-fold higher uptake of [^18^F]F-DcFPYL in mice submandibular and sublingual glands compared to that of rats. Additional in vitro autoradiography studies on human, mouse, and rat salivary glands showed similar binding of [^18^F]F-DcFPYL for the mouse and human salivary glands. Therefore, they argued that this mouse model is a more suitable research model for PSMA-TRT compared to the rat. However, since the relative uptake of the PSMA-targeted tracer in the murine salivary glands in this study was not comparable to the high uptake in the human salivary glands, it is still questionable whether the NCR-nu/nu mice represent an appropriate research model to examine PSMA-TRT toxicity effects.

Despite the lack of preclinical models that adequately resemble human salivary gland uptake of PSMA-targeting tracers, a few studies reported on protective strategies for the salivary glands and/or kidneys. Rousseau et al. used LNCap-tumor bearing NOD SCID gamma (NSG) mice to demonstrate that administration of monosodium glutamate (MSG), a well-known food additive, can reduce uptake of [^68^Ga]Ga-PSMA-11 in the salivary glands and kidneys without affecting uptake in the tumor. Because the majority of PSMA ligands integrate glutamate to bind to PSMA, the administration of MSG may act by blocking this process and thereby decreasing non-specific binding in healthy organs [122].

Another strategy that was tested, was the use of 2-(phosphonomethyl)pentane-1,5-dioic acid (2-PMPA), a well-known PSMA inhibitor which is frequently used as a competitor for PSMA-targeted tracers [123]. It was shown that administration of 2-PMPA decreased binding of PSMA-targeted tracers in the kidneys in a dose dependent manner, but at the expense of (to a lesser extent) decreased uptake in the tumor [31,113,116]. In line of this research it has been reported that slightly decreasing the molar activity, or the using low concentrations of unlabeled or non-radioactive labeled tracers can positively influence the tumor-to-kidney-ratio and warrants further research [113,124,125]. In order to specifically shield the kidneys and salivary glands from PSMA-TRT with 2-PMPA, a novel prodrug (e.g., Tris-POC-2-PMPA) has been proposed which is suggested to specifically bind in non-malignant tissues and hence reduce toxicity [126]. Further (pre)clinical research is needed to investigate the potential of this compound to protect the salivary glands and kidneys without affecting tumor uptake.

Finally, a strategy to reduce renal uptake of PSMA-targeted tracers is the use of mannitol, which can act as an osmotic diuretic in the PSMA expressing proximal tubules of the kidneys. Because mannitol promotes diuresis, reabsorption of the PSMA-targeting tracers may decrease, which is hypothesized to decrease overall renal uptake [127]. However, first clinical imaging studies revealed that mannitol did not show a significant effect on the uptake of PSMA-specific tracers [108,127].

To summarize, it has become evident that there is an urgent need for PSMA-specific renal and salivary gland protection for PCa patients receiving PSMA-TRT. The lack of an adequate preclinical research model to reflect the situation in humans is a major challenge. Increasing evidence suggests that besides PSMA-specific binding, also non-specific binding of PSMA may play a role in the uptake of PSMA-targeting tracers in the salivary glands. Further investigations are needed to identify a compound that is able to selectively block both specific and/or non-specific binding of PSMA tracers in the salivary glands and the kidneys to improve safety without affecting tumor uptake.

## 7. Concluding Remarks

PSMA-targeting tracers have made a steep introduction into clinical staging of PCa and a fast implementation towards PSMA-TRT. This fast clinical integration, however, requires preclinical research to assure optimal use with maximal safety. Preclinical PSMA-TRT studies are able to test and compare a wide range of methods in a high throughput manner in order to ultimately improving PSMA-TRT therapeutic effectiveness while simultaneously decreasing the uptake in the healthy PSMA-expressing kidneys and salivary glands. Improving already existing PSMA-targeting tracers, creating bi-ligands, experimenting with various radionuclides, enhancing therapeutic effects by using radio-sensitizers, and looking for compounds that selectively block the healthy PMSA-expressing organs from PSMA-TRT are major focus points in PSMA preclinical research. The preclinical studies discussed here and ongoing preclinical research will inevitably determine the future of PSMA-TRT in the clinic.

## Figures and Tables

**Table 1:** Overview of PSMA-targeting small molecule inhibitors suitable for therapy.

Compound	Radionuclides	PSMA Binding Motif	First Reference	Clinical Trials
PSMA-617	Lutetium-177 Actinium-225 Indium-111 Gallium-68 Yttrium-90	Urea based (Glu-urea-lys)	[26]	[27,28]
PSMA-I&T	Lutetium-177 Actinium-225 Indium-111 Gallium-68	Urea based (Glu-urea-lys)	[29]	[30]
MIP-1095	Iodine-123 Iodine-131	Urea based (Glu-urea-lys)	[31]	[32]
MIP-1072	Iodine-123	Urea based (Glu-urea-lys)	[31]	[33]
MIP-1404/-1405	Technetium-99m	Urea based (Glu-urea-lys)	[34]	[35]
PSMA I&S	Technetium-99m	Urea based (Glu-urea-lys)	[36]	-
CTT1400/CTT1402	Lutetium-177	phosphoramidate-based	[37]	-
RPS-027	Iodine-131 Astatine-211	Urea based (Glu-urea-lys)	[38]	-
DCIBzL	Iodine-125 Iodine-131	Urea based (Glu-urea-lys)	[39]	-

**Table 2:** Overview of PSMA-targeting small molecule inhibitors that have been conjugated with an albumin binding domain and their in vivo biodistribution data in mice. * Values had to be based on graphs rather than exact data as they were not present in the reference.

Compound	Radionuclide	Albumin Binding Domain	In Vivo Model	Injected	Tumor-to-kidney-Ratio 24 h p.i.	Ref.
CTT1402	Lutetium-177	4-(*p*-iodophenyl) butyric acid	NCr nude mice + PC3-PIP tumors	1.85 ± 0.07 MBq 4 ± 1 MBq/nmol	0.14	[37]
RPS-027	Iodine-131	4-(4-iodophenyl) butanoic acid	NCr-nu/nu mice + LNCaP cell xenografts	∼370 kBq/10 μCi	±2.5 *	[38]
DOTA-PSMA-ALB-02	Lutetium-177	4-(*p*-iodophenyl) butyric acid	PC-3 PIP/flu	No data	7.16	[50]
HTK01169	Lutetium-177	*N*-[4-(*p*-iodophenyl)butanoyl]-Glu	LNCaP	No data	0.45	[51]
DOTA-EB-MCG	Yttrium-90	truncated Evans blue	Athymic Nude-Foxn1nu, Envigo + PC3-PIP tumors	3.7–5.1 MBq	±4 *	[46]
RPS-072	Lutetium-177	4-(4-iodophenyl) butanoic acid	Male BALB/C athymic nu/nu mice + LNCaP tumors	0.36–1.3 MBq 13–23 pmol	±4.5 *	[53]
RPS-074	Actinium-225	4-(4-iodophenyl) butanoic acid	Male BALB/C athymic nu/nu mice + LNCaP tumors	105 kBq 142 pmol	4.3	[54]
PSMA-ALB-53	Lutetium-177	4-(*p*-iodophenyl)-moiety	PC3-PIP tumors	5MBq 100 pmol	2.17	[52]
PSMA-ALB-56	Lutetium-177	*p*-(tolyl)-moiety	PC3-PIP tumors	5MBq 100 pmol	10.8	[52]

**Table 3:** Overview of radionuclides used in PSMA-TRT (pre)clinical research and their basic characteristics.

Radionuclide	Particle Emission	T_1/2_	*Eavg KeV* (β^−^ or α)
Scandium-47	β^−^	3.3 days	162
Copper-67	β^−^	2.6 days	141
Iodine-131	β^−^	8.0 days	181
Terbium-161	β^−^	6.9 days	154
Lutetium-177	β^−^	6.7 days	140
Astatine-211	α	7.2 h	5868
Lead-212	β^−^	10.6 h	130
Bismuth-213	α	46 min	1390 (max)
Actinium-225	α	9.9 days	5915
Thorium-227	α	18.7 days	6145
